# Preconditioning with selective autoretroperfusion: *In vivo* and *in silico* evidence of washout hypothesis

**DOI:** 10.3389/fbioe.2024.1386713

**Published:** 2024-05-10

**Authors:** Jenny S. Choy, Terry Hubbard, Haifeng Wang, Yousif Awakeem, Pouya Khosravi, Bahram Khadivi, Jose A. Navia, Gregg W. Stone, Lik Chuan Lee, Ghassan S. Kassab

**Affiliations:** ^1^ California Medical Innovations Institute, Inc., San Diego, CA, United States; ^2^ 3DT Holdings, LLC, San Diego, CA, United States; ^3^ Department of Mechanical Engineering, Michigan State University, East Lansing, MI, United States; ^4^ Scripps Memorial Hospital, Division of Cardiology, La Jolla, CA, United States; ^5^ Department of Surgery, Austral University, Buenos Aires, Argentina; ^6^ Division of Cardiology, Department of Medicine, Icahn School of Medicine at Mount Sinai, The Zena and Michael A. Wiener Cardiovascular Institute, New York, United States

**Keywords:** STEMI, retroperfusion, reperfusion injury, preconditioning, computational modeling, washout

## Abstract

**Introduction:**

Prompt reperfusion of coronary artery after acute myocardial infarction (AMI) is crucial for minimizing heart injury. The myocardium, however, may experience additional injury due to the flow restoration itself (reperfusion injury, RI). The purpose of this study was to demonstrate that short preconditioning (10 min) with selective autoretroperfusion (SARP) ameliorates RI, based on a washout hypothesis.

**Methods:**

AMI was induced in 23 pigs (3 groups) by occluding the left anterior descending (LAD) artery. In SARP-b (SARP balloon inflated) and SARP-nb (SARP balloon deflated) groups, arterial blood was retroperfused for 10 min via the great cardiac vein before releasing the arterial occlusion. A mathematical model of coronary circulation was used to simulate the SARP process and evaluate the potential washout effect.

**Results:**

SARP restored left ventricular function during LAD occlusion. Ejection fraction in the SARP-b group returned to baseline levels, compared to SARP-nb and control groups. Infarct area was significantly larger in the control group than in the SARP-b and SARP-nb groups. End-systolic wall thickness was preserved in the SARP-b compared to the SARP-nb and control groups. Analyte values (pH, lactate, glucose, and others), measured every 2 min during retroperfusion, suggest a “washout” effect as one important mechanism of action of SARP in reducing infarct size. With SARP, the values progressively approached baseline levels. The mathematical model also confirmed a possible washout effect of tracers.

**Discussion:**

RI can be ameliorated by delaying restoration of arterial flow for a brief period of time while pretreating the infarction with SARP to restore homeostasis via a washout mechanism.

## Graphical Abstract

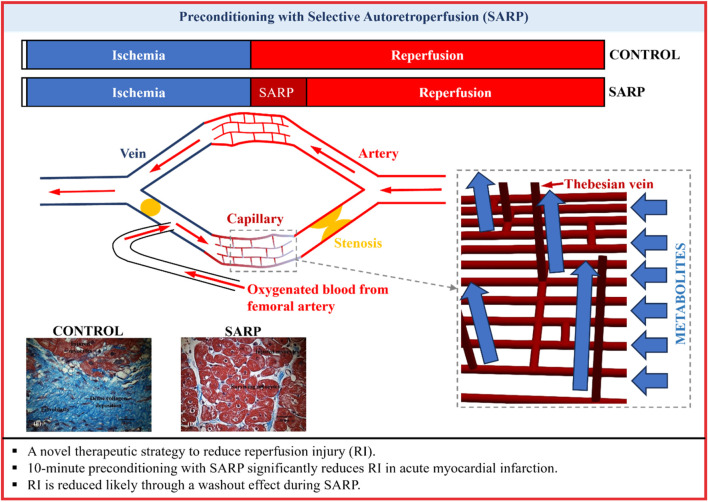

## Introduction

Heart disease is the primary cause of cardiovascular mortality (Virani et al., 2021) in the United States for men, women, and people of most racial and ethnic groups (Centers for Disease Control and Prevention, 2018). Coronary artery disease is the most common type of heart disease, killing 360,900 people in 2019 (Virani et al., 2021). Every year, more than 800,000 Americans suffer an acute myocardial infarction (AMI) (Fryar et al., 2012), more than 600,000 corresponding to a first episode (Fryar et al., 2012). ST-segment-elevation myocardial infarction (STEMI) is the most severe form of AMI, causing significant morbidity and mortality (Vogel et al., 2019), and imposing heavy clinical and financial burden on the American healthcare system. Primary percutaneous coronary intervention (PCI) and thrombolytic therapy are effective in treating STEMI (Welch et al., 2012; Frampton et al., 2020; Valgimigli and Gragnano, 2020), reducing hospitalizations and long-term mortality. Many patients, however, experience long-term cardiac reperfusion issues due to lethal arrhythmias, myocardial necrosis, microvascular dysfunction (no reflow phenomenon), stunning myocardium, and heart failure (Verma et al., 2002; Papageorgiou et al., 2018), as a result of ischemia reperfusion injury (RI) (Yellon and Hausenloy, 2007; Hausenloy and Yellon, 2013; Davidson et al., 2019) and distal embolization (De Maria et al., 2013). Hence, there is a need for practical modalities of treatment to limit the extent of RI without first requiring opening the arterial obstruction.

Unlike the obstructed coronary arterial system, the coronary venous system remains healthy and unobstructed, and thus has enormous potential for therapy delivery (Katti and Patil, 2012; Rodenberg et al., 2019), including retrograde delivery of arterial blood (Kassab et al., 1985). Although envisioned a century ago (Pratt, 1898; Beck and Stanton, 1948), therapeutic retroperfusion (a type of coronary sinus intervention), has not been adopted clinically because complicated equipment has been required to regulate perfusion to prevent damage to the coronary venous system when exposed to arterial pressures. Synchronized retrograde perfusion and pressure-controlled intermittent coronary sinus occlusion are retroperfusion methods used to treat myocardial ischemia. These techniques, combined with a balloon-tipped catheter, redirect coronary sinus blood or pump arterial blood during diastole to the ischemic myocardium. They reduce infarct size, myocardial hemorrhage, and no-reflow phenomenon (Mohl, 1988; Mohl et al., 1988; Wakida et al., 1993), and improve left ventricular (LV) function (Yamazaki et al., 1985). Their widespread use, however, has been limited due to safety concerns and the need for repeated occlusion of the coronary sinus (Aldea et al., 1996; Lazar, 2004). Our group has been investigating for several years the use of retroperfusion (selective autoretroperfusion, SARP) for ischemic myocardium without the need of complicated apparatuses to regulate pressure in the cardiac venous system (Choy et al., 2011; Choy et al., 2020). We have demonstrated that SARP, alone or in combination with mild-hypothermia was able to confer cardiac protection following occlusion of a coronary artery by reducing infarct size in a swine model of anterior left ventricular (LV) myocardial infarction (Choy et al., 2020). The present study demonstrates that SARP can be accomplished safely with a catheter-based, pressure-regulated (∼60 mmHg, using the animal’s own pulse pressure) method of selective (left ventricular anterior wall) delivery of arterial blood to the ischemic myocardium to substantially reduce the deleterious effects of reperfusion. This true preconditioning of the myocardium for a brief duration is hypothesized to substantially reduce RI. We demonstrated the beneficial effects of preconditioning STEMI with SARP for 10 min before opening the arterial obstruction to combat RI. The results presented here, make SARP a very appealing modality of treatment for mitigation of RI.

## Materials and methods

### *In-vivo* experiments

All animal experiments were performed in accordance with national and local ethical guidelines, including the ARRIVE guidelines, the Guide for the Care and Use of Laboratory Animals, the Public Health Service Policy on Humane Care and Use of Laboratory Animals, and the Animal Welfare Act, and an approved California Medical Innovations Institute IACUC protocol, 053.1, regarding the use of animals in research. Patients were not involved in this study.

#### Animal preparation

Twenty three (n = 23) female Yorkshire domestic swine with body weight of 57.4 ± 3.6 kg were blindly randomized in three groups, SARP-balloon (SARP-b, n = 6), SARP-no balloon (SARP-nb, n = 5), and control (n = 12). All three groups received coronary arterial occlusion. In addition, both the SARP-b and SARP-nb groups received retroperfusion. In the SARP-b group, the SARP balloon at the distal tip was inflated to avoid anterograde flow towards the coronary sinus, while in the SARP-nb group it was not inflated. After overnight fasting, sedation was achieved with TKX (Telazol 10 mg/kg, Ketamine 5 mg/kg, and Xylazine 5 mg/kg, IM), and surgical anesthesia was maintained with isoflurane 1.5%–2%. Ventilation with oxygen was provided with a respirator and maintained PCO_2_ at approximately 35–40 mmHg. Electrocardiographic leads were attached to the animal’s limbs to monitor the electrical activity of the heart. Body temperature was kept at 36.0°C–37.5°C with a warming blanket. Introducer sheaths were placed in the right jugular vein (for administration of saline and drugs, and advancement of the SARP catheter towards the coronary sinus) and both femoral arteries (for advancement of the percutaneous transluminal coronary angioplasty (PTCA) balloon catheter into the left anterior descending (LAD) artery, and procurement of arterial blood for retroperfusion). The animals were heparinized (100 IU/kg, IV) before further instrumentation.

#### Ischemic model

The animals were premedicated with Amiodarone, 25 mg diluted in 10 mL of dextrose every 5 min, 5 times, in order to control arrhythmias. An over-the-wire PTCA balloon catheter was inserted through the right femoral artery and positioned under fluoroscopic guidance into the LAD artery, distal to the second diagonal branch. The exact same location for balloon placement was selected in all three groups to create approximately similar areas of ischemia in all the animals. Preconditioning of the artery was performed by inflating and deflating the balloon for 30 s, 3 times, to make the myocardium more resistant to a greater ischemic insult. The balloon was then inflated for 70 min in the SARP-b and SARP-nb groups (the last 10 min in sync with retroperfusion), and 60 min in the control group. All procedures were strictly controlled by the study director.

#### Selective autoretroperfusion (SARP)

The SARP catheter was inserted through the right jugular vein, advanced into the coronary sinus, and positioned in the great cardiac vein (GCV) at the coronary sulcus. The catheter was then connected to the arterial sheath in the left femoral artery via silicone tubing attached to a custom-made connector, and the air was purged. A flow sensor was positioned around the silicone tubing to measure blood flow. A pressure guide wire was advanced through the SARP catheter towards the distal tip. With all catheters in place, baseline measurements (echocardiography, blood sample collection, arterial pressure, and ECG recording) were taken before initiation of the procedure.

Arterial blood, shunted from the left femoral artery, passed through the silicone tubing, and was then delivered to the GCV via the SARP catheter. The arterial blood was delivered into the GCV using the animal’s own pulse pressure (i.e., autoretroperfusion) without the need of synchronized pumps. Norepinephrine, 2–4 μg/min, IV was administered to compensate for the decrease in pressure due to the LAD occlusion. Retroperfusion was performed during the last 10 min of occlusion in the SARP-b and SARP-nb groups, after which, the occlusion was released and the animals were recovered for 4 weeks. Echocardiography, blood sampling, and *ex-vivo* heart preparation are described in the Supplementary Material section.

#### Statistical analysis

All statistical analyses were performed using SPSS v26 (IBM Corp, Armonk, NY). The data were expressed as mean ± standard error (SE). The differences between the various parameters and groups were evaluated using a mixed model ANOVA design, with a Between Subjects variable (Group: SARP-b, SARP-nb, and Control) and a Within Subjects variable (Time). When an interaction between Group and Time was found, simple main effects were examined to look at group differences within each time point and to look at time differences within each group. The differences were considered significant at *p* < 0.05, and a Bonferroni correction was used to cap the familywise error at 5%.

### Mathematical model

A validated computer model of the coronary artery-capillary-vein network and an indicator-tracking approach (Wang et al., 2024) (Supplementary Material) were used to test the washout mechanism of SARP. Briefly, this computer model considers the coronary arteries, veins (including Thebesian veins), and capillaries, as well as their interactions with the heart in a closed-loop system (Supplementary Figure S1). In total, this network contains 3,683 blood vessels. Each of these vessels is represented by a mathematical (nonlinear lumped-parameter) model that considers their size (diameter and length), mechanical properties, and how they branch off from one another. Model parameters are prescribed in Supplementary Tables S1–S4. Flow resistance and capacitance (compliance) for vessels of each order are scaled based on the measured branching ratios in pigs. The peripheral circulation is represented by a linear lumped-parameter model. Contraction of the heart’s left ventricle (LV) and left atrium (LA) is represented by a time-varying elastance model. A microvascular network consisting of the small arteries, veins, and capillaries (order 6 to −6) is embedded in the coronary circulation (Supplementary Figure S1). The effects of 15 additional equivalent microvascular networks are also simulated in this computer model, by matching the pressures at the entry (*P*_*i*_) and exit (*P*_*o*_) points of each equivalent microvascular network with those at the entry and exit points of the embedded microvascular network. Thebesian veins are randomly distributed between arterioles and venules. Vessel order numbers are assigned based on diameter using the diameter-defined Strahler system. The smallest arteries are designated as vessels of order 1 when two capillaries (order 0) meet; larger arteries are designated as vessels of orders 1, 2, … , 11 in the direction of increasing vessel diameter. Veins follow the same rules as arteries but are numbered with a negative sign, ranging from −1 to −12 in increasing diameter order. To simulate the SARP procedure, the large coronary artery and the distal GCV are occluded, as indicated by the black crosses, and pulsatile femoral pressure is applied to the area proximal to the GCV (Supplementary Figure S1).

The indicator-tracking approach (Wang et al., 2024) was used to evaluate the efficiency of metabolite washout from occluded regions to the heart’s chambers via Thebesian veins, in the context of SARP. In this method, indicators (representing metabolites) are “virtually” released at various locations (e.g., arterioles of order 1, a larger artery of order 7, or a vein of order −7). The indicators are monitored over a duration of 10 min, allowing the assessment of temporal and spatial movement of indicators (metabolites). The two methods of indicator release are outlined in the Supplementary Material section.

The model parameters are determined and calibrated based on our previous studies (Fan et al., 2020; Fan et al., 2021; Wang et al., 2024). Using this set of model parameters, the predictive accuracy of the model for blood pressure and flow rate distributions has been validated through comparison with other computational studies (Wang et al., 2024). The particle-tracking approach used in the computational model has also been validated against experimental measurements (Wang et al., 2024).

## Results

This study investigated the efficacy of short-term treatment of STEMI with selective autoretroperfusion (SARP) before coronary artery reperfusion. SARP mitigated the deleterious effects of reperfusion injury (RI), preserving cardiac function and reducing myocardial infarct size, suggesting a potential washout mode of action.

Table 1 summarizes the left ventricular diameter and wall thickness in end-systole and end-diastole, measured from echocardiographic images during baseline, ischemia, retroperfusion, and 4 weeks reperfusion. End-systolic wall thickness was significantly larger in the SARP-b group (0.84 ± 0.04 cm) than in the SARP-nb (0.71 ± 0.04 cm, *p* < 0.05) and control (0.70 ± 0.03, *p* < 0.05) groups during retroperfusion. After 4 weeks of reperfusion, end-systolic wall thickness was similar to baseline in the SARP-b group (0.79 ± 0.04) and larger than in the SARP-nb (0.67 ± 0.02, *p* < 0.05) and control (0.59 ± 0.03, *p* < 0.01) groups.

**TABLE 1:** Left ventricular diameter and wall thickness.

LVEDD (mm)	SARP-b	SARP-nb	Control
Baseline	42.46 ± 1.46	45.48 ± 1.27	42.71 ± 0.90
Ischemia	44.61 ± 1.66	46.59 ± 1.20	44.26 ± 0.71
Retroperfusion	41.89 ± 1.02	45.46 ± 1.52	43.27 ± 1.02
Four weeks reperfusion	44.07 ± 2.11	46.19 ± 1.50	44.62 ± 0.58

LVESD (mm)	SARP-b	SARP-nb	Control
Baseline	30.69 ± 1.26	32.12 ± 0.91	28.91 ± 1.38
Ischemia	31.92 ± 1.31	33.85 ± 1.13	30.99 ± 0.44
Retroperfusion	26.76 ± 1.24	28.54 ± 1.58	29.70 ± 0.78
Four weeks reperfusion	28.57 ± 1.93	30.66 ± 1.01	29.95 ± 0.85

LVEDWT (cm)	SARP-b	SARP-nb	Control
Baseline	0.64 ± 0.03	0.56 ± 0.03	0.58 ± 0.02
Ischemia	0.60 ± 0.04	0.56 ± 0.05	0.54 ± 0.02
Retroperfusion	0.59 ± 0.06	0.57 ± 0.05	0.55 ± 0.06
Four weeks reperfusion	0.65 ± 0.05	0.56 ± 0.03	0.53 ± 0.03

LVESWT (cm)	SARP-b	SARP-nb	Control
Baseline	0.74 ± 0.04	0.69 ± 0.03	0.69 ± 0.02
Ischemia	0.75 ± 0.04	0.65 ± 0.05	0.68 ± 0.06
Retroperfusion	0.84 ± 0.04Ɨ‡	0.71 ± 0.04	0.70 ± 0.03
Four weeks reperfusion	0.79 ± 0.04Ɨ§	0.67 ± 0.02	0.59 ± 0.03*

LVEDD, left ventricular end-diastolic diameter; LVESD, left ventricular end-systolic diameter; LVEDWT, left ventricular end-diastolic wall thickness; LVESWT, left ventricular end-systolic wall thickness. *p < 0.05 relative to baseline, ‡*p* < 0.05 relative to control, §*p* < 0.01 relative to control.

Left ventricular global and segmental longitudinal strains are summarized in Tables 2, 3, respectively. The global strain values in all three groups were similar at baseline (SARP-b = −21.4% ± 0.8%, SARP-nb = −22.6% ± 1.2%, Control = −21.7% ± 0.8%; *p* > 0.05) and ischemia (SARP-b = −13.5% ± 1.1%, SARP-nb = −13.6% ± 2.3%, Control = −12.7% ± 1.4%; *p* > 0.05). In the SARP-b group, the global strain decreased ∼40% during ischemia, *p* < 0.001, but recovered to similar baseline values during retroperfusion (−19.4% ± 0.9%, *p* > 0.05) and 4-weeks reperfusion (−19.1% ± 0.7%, *p* > 0.05). In the SARP-nb group, the global strain also decreased ∼40% during ischemia, *p* < 0.05 and recovered to baseline values during retroperfusion (−19.6% ± 1.7%, *p* > 0.05) and 4-weeks reperfusion (−20.6% ± 1.2%, *p* > 0.05). In the control group, however, the strain decreased ∼40% during the first half of ischemia, *p* < 0.001, continued decreasing during the second half (−11.7% ± 0.9%, *p* < 0.001), and remained almost at the same levels during 4-weeks retroperfusion (−13.1% ± 1.2%, *p* < 0.001). Table 3 shows the segmental longitudinal strain at basal, middle, and apical interventricular septum, and basal, middle, and apical anterolateral wall in all three groups.

**TABLE 2:** Comparison of left ventricular global longitudinal strain.

	SARP-b	SARP-nb	Control
Baseline	−21.4 ± 0.8	−22.6 ± 1.2	−21.7 ± 0.8
Ischemia	−13.5 ± 1.1^b^	−13.6 ± 2.3^a^	−12.7 ± 1.4^b^
Retroperfusion	−19.4 ± 0.9^d^	−19.6 ± 1.7^c^	−11.7 ± 0.9^b^
Four weeks reperfusion	−19.1 ± 0.7^c^	−20.6 ± 1.2^c^	−13.1 ± 1.2^b^

With respect to baseline in their corresponding group, the *p*-values were, ^a^ *p* < 0.05, ^b^ *p* < 0.001. With respect to Control, the *p*-values were, ^c^ *p* < 0.01, ^d^ *p* < 0.001.

**TABLE 3:** Comparison of left ventricular longitudinal strain per segments.

SARP-b	BIS	MIS	AIS	BAL	MAL	AAL
Baseline	−13.5 ± 0.5	−21.7 ± 2.4	−24.1 ± 1.5	−19.9 ± 2.3	−20.6 ± 1.9	−28.3 ± 0.9
Ischemia	−8.6 ± 1.3^b^	−13.5 ± 1.1^a^	−16.9 ± 1.3^b^	−12.4 ± 2.0^a^	−10.6 ± 1.6^b^	−19.0 ± 2.2^b^
Retroperfusion	−13.5 ± 1.6	−19.9 ± 1.2	−19.4 ± 1.6	−20.4 ± 2.5	−19.2 ± 1.7	−24.0 ± 2.6
Four weeks reperfusion	−11.6 ± 0.7	−20.9 ± 1.8	−18.6 ± 3.0	−21.4 ± 2.7	−16.5 ± 1.2	−25.5 ± 1.3

SARP-nb	BIS	MIS	AIS	BAL	MAL	AAL
Baseline	−16.9 ± 2.9	−21.6 ± 1.2	−26.4 ± 2.0	−21.1 ± 6.7	−20.3 ± 2.2	−29.2 ± 3.0
Ischemia	−7.3 ± 1.1^a^	−14.2 ± 2.3^a^	−15.5 ± 1.9^b^	−15.6 ± 2.4	−11.1 ± 3.5	−18.0 ± 4.4
Retroperfusion	−13.1 ± 2.1	−18.6 ± 3.0	−21.0 ± 2.8	−23.7 ± 2.3	−18.1 ± 3.4	−23.2 ± 2.5
Four weeks reperfusion	−16.1 ± 2.7	−18.8 ± 0.8	−19.9 ± 1.3	−23.0 ± 1.9	−19.7 ± 1.8	−25.9 ± 4.5

Control	BIS	MIS	AIS	BAL	MAL	AAL
Baseline	−13.2 ± 1.1	−24.3 ± 1.5	−23.9 ± 1.4	−20.2 ± 2.1	−18.0 ± 0.5	−30.7 ± 2.8
Ischemia	−7.6 ± 1.1^b^	−6.4 ± 6.3^a^	−13.9 ± 0.7^c^	−16.6 ± 2.6	−12.1 ± 2.4	−19.5 ± 1.9^b^
Ischemia	−4.1 ± 3.1^a,d^	−11.5 ± 1.9^c,e^	−12.5 ± 0.7^c,e^	−15.9 ± 2.1	−9.2 ± 1.5^b,f^	−16.9 ± 1.1^b,d^
Four weeks reperfusion	−6.8 ± 1.1^c,e^	−12.8 ± 2.1^c,e^	−14.1 ± 1.0^c^	−18.8 ± 1.7	−10.5 ± 1.6^b,d^	−15.9 ± 1.8^b,e^

BIS, basal interventricular septum; MIS, middle interventricular septum; AIS, apical interventricular septum; BAL = basal anterolateral wall; MAL, middle anterolateral wall; AAL = apical anterolateral wall. With respect to baseline in their corresponding group, the *p*-values were, ^a^ *p* < 0.05, ^b^ *p* < 0.01, ^c^ *p* < 0.001. With respect to SARP-b, the *p*-values were, ^d^ *p* < 0.05, ^e^ *p* < 0.01, ^f^ *p* < 0.001.

**FIGURE 1:**
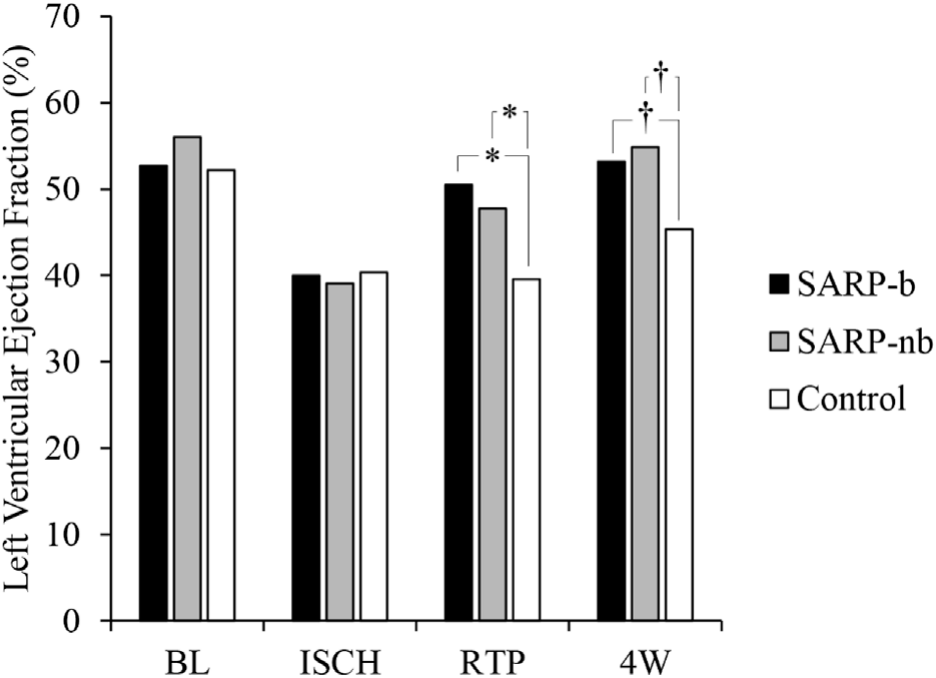
Changes in Left Ventricular Ejection Fraction (LVEF) in the SARP-b, SARP-nb, and control groups at different time points. BL = Baseline, ISCH = Ischemia, RTP = Retroperfusion, and 4W = Four weeks. * = *p* < 0.001 and † = *p* < 0.01 between cohorts.

Left ventricular function, assessed by measuring ejection fraction (LVEF) with echocardiography, is shown in Figure 1. There was significant interaction between Group (SARP-b, SARP-nb, and Control) and Time (baseline, ischemia, retroperfusion, and 4 weeks), (F_(4.5, 45.4)_ = 9.4, *p* < 0.001), therefore simple main effects at each time point were analyzed. There was no significant difference between the three groups at baseline (F_(2, 20)_ = 3.3, *p* > 0.05) and during ischemia (F_(2, 20)_ = 0.2, *p* = NS), but there were significant differences in LVEF during retroperfusion (F_(2, 20)_ = 26.7, *p* < 0.001) and at 4 weeks (F_(2, 20)_ = 11.5, *p* < 0.001). At retroperfusion, Bonferroni *post hoc* tests showed that both the SARP-b (50.5% ± 1.3%) and SARP-nb (47.7% ± 1.9%) groups had significantly higher LVEF than the control group (39.6% ± 0.8%, *p* < 0.001 for both comparisons), and there was no statistically significant difference in LVEF between the SARP-b and SARP-nb groups. At 4 weeks, Bonferroni *post hoc* tests showed that both the SARP-b (53.2% ± 2.2%) and the SARP-nb (54.9% ± 1.7%) groups had significantly higher LVEF than the control group (45.4% ± 1.1%, *p* < 0.01 for both comparisons), and there was no statistically significant difference in LVEF between the SARP-b and SARP-nb groups.

**FIGURE 2:**
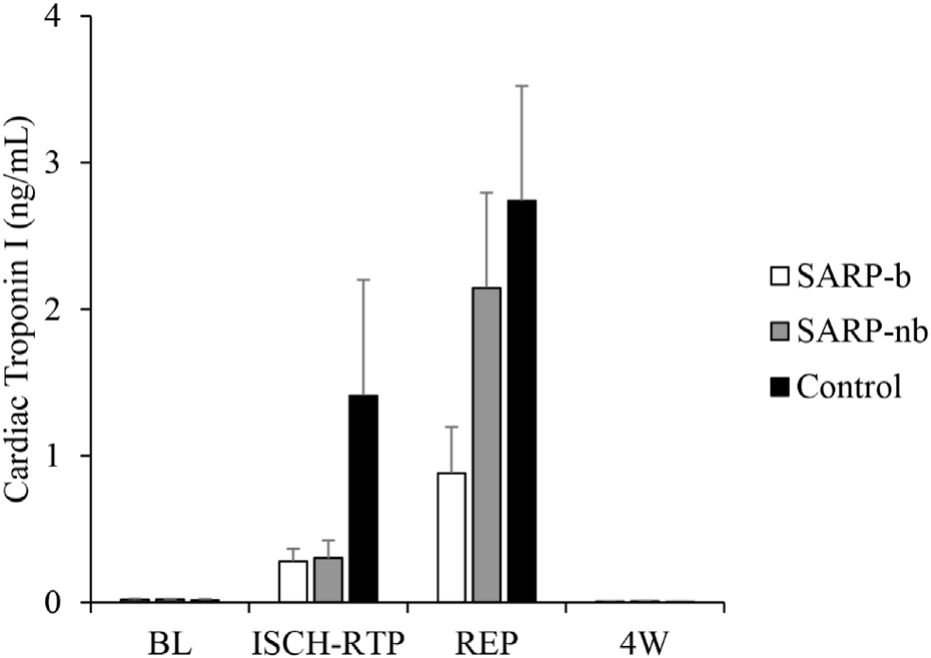
Cardiac Troponin I levels at different time points in the SARP-b, SARP-nb, and control groups. BL = Baseline, ISCH-RTP = Ischemia-Retroperfusion, REP = Reperfusion, and 4W = Four weeks.

Figure 2 shows cardiac troponin (cTnI) values at different time points in all three cohorts. There were significant differences within each group over time. There was no significant interaction, however, between Group and Time. The levels of cTnI were similar (<0.03 ng/mL) in all three groups at baseline. They increased to 0.28 ± 0.09 ng/mL during ischemia-retroperfusion in the SARP-b group, 0.30 ± 0.12 ng/mL in the SARP-nb group, and 1.41 ± 0.79 ng/mL in the control group. Although cTnI in the control group was higher than in the SARP-b and SARP-nb groups, the values in the control group during ischemia-retroperfusion were not significantly different relative to baseline due to intragroup variability. During reperfusion, the values in all three groups continued to increase, 0.88 ± 0.31 ng/mL in the SARP-b group, 2.48 ± 0.76 ng/mL in the SARP-nb group, and 2.74 ± 0.78 ng/mL in the control.

**FIGURE 3:**
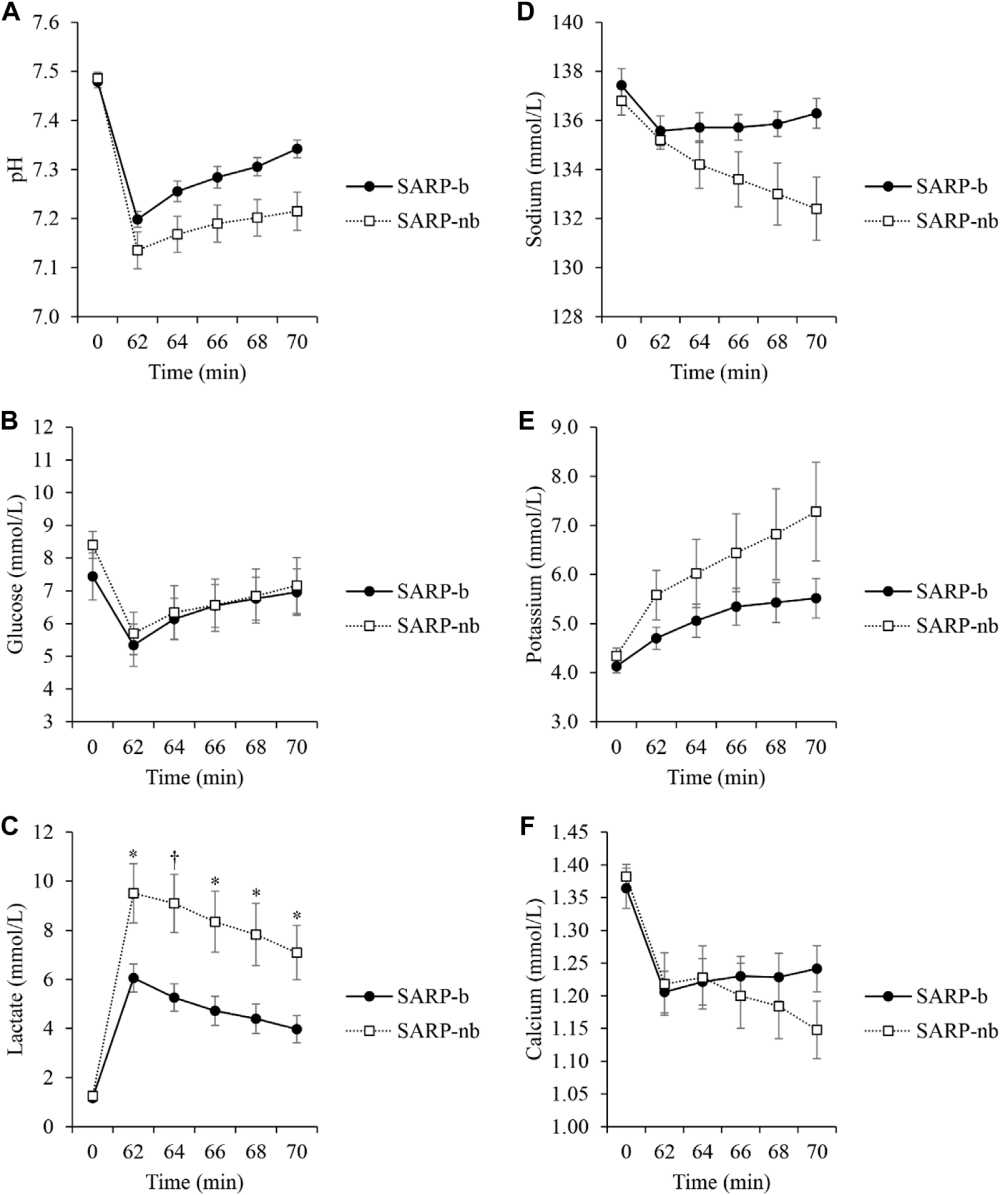
Changes in several analytes: pH **(A)**, Glucose **(B)**, Lactate **(C)**, Sodium **(D)**, Potassium **(E)**, and Calcium **(F)** during 10 min of retroperfusion in the SARP-b and SARP-nb groups. Blood was obtained from the lumen of the PTCA balloon catheter occluding the Left Anterior Descending artery. * = *p* < 0.05 and † = *p* < 0.01 between cohorts.

Figure 3 shows the values of several analytes like pH (Figure 3A), glucose (Figure 3B), lactate (Figure 3C), sodium (Figure 3D), potassium (Figure 3E), and calcium (Figure 3F) measured every 2 min from effluent blood samples obtained through the lumen of the PTCA balloon catheter that was occluding the LAD artery. The values of the corresponding analytes were similar in both groups at baseline (*p* = NS). As shown in the plots (Figure 3), the values in the SARP group progressively got closer to baseline values as retroperfusion progressed from minute 62 to minute 70 while the LAD artery was still occluded. There was significant interaction between Group and Time for lactate (F_(1.6,16.1)_ = 6.2, *p* < 0.05), therefore simple main effects at each time point were analyzed. There was no significant difference between the SARP-b and SARP-nb groups at baseline (F_(1,10)_ = 0.1, *p* > 0.05), but there were significant differences in lactate levels at minutes 62 (F_(1,10)_ = 8, *p* < 0.05), 64 (F_(1,10)_ = 10, *p* < 0.01), 66 (F_(1,10)_ = 8, *p* < 0.05), 68 (F_(1,10)_ = 7, *p* < 0.05), and 70 (F_(1,10)_ = 8, *p* < 0.05). All the other analytes showed significant differences within each group over time but no interaction between the two groups.

**FIGURE 4:**
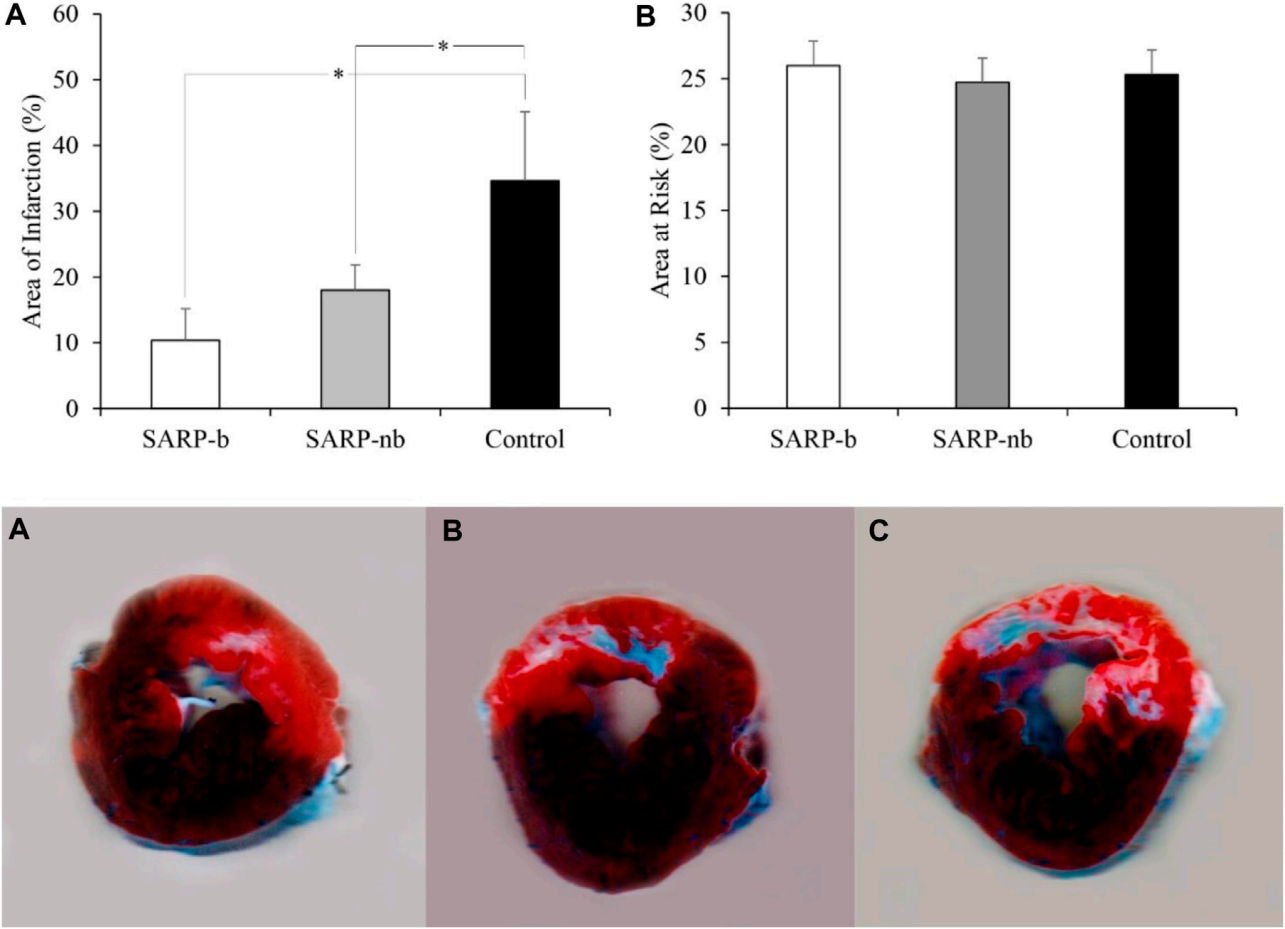
Upper panel. **(A)** Area of Infarction expressed as the percentage of the area at risk. SARP-b and SARP-nb were significantly different than control (*p < 0.05 between both comparisons). Upper panel **(B)** Area at Risk expressed as the percentage of the LV area. Both the area of infarction and the area at risk were calculated from myocardial sections double-stained with Evans blue and triphenyl tetrazolium chloride in the SARP-b, SARP-nb, and control groups. Lower panel. Myocardial sections from SARP-b **(A)**, SARP-nb **(B)**, and Control **(C)** groups demarcating the area of infarction (white), the area at risk (red), and the non-affected area (blue).

The area of infarction, expressed as the percentage of the area at risk, is shown in Figure 4 (upper panel, A). The infarct area in the SARP-b group was significantly smaller than in the control group (10.4% ± 4.8% vs. 34.7% ± 10.5%, *p* < 0.05). Similarly, the infarct area in the SARP-nb group was significantly smaller compared to the control group (18.0% ± 3.8%, *p* < 0.05). There was no significant difference between the SARP-b and SARP-nb groups. The area at risk, expressed as the percentage of the total LV area, is also shown in Figure 4 (upper panel, B). The area at risk was similar in all three groups, SARP-b, SARP-nb, and control (26.0% ± 1.9%, 24.7% ± 2.4%, and 25.3% ± 0.7%, respectively, *p* > 0.05). The lower panel shows representative images of myocardial slices double-stained with Evans blue and triphenyl tetrazolium chloride, demonstrating the area of infarction (white), the area at risk (red), and the non-affected area (blue) in the A) SARP-b, B) SARP-nb, and C) control groups. The area of infarction extended throughout the full thickness of the myocardial wall in the control group, and to a lesser degree in the SARP-nb group, whereas in the SARP-b group it was scattered throughout the wall.

**FIGURE 5:**
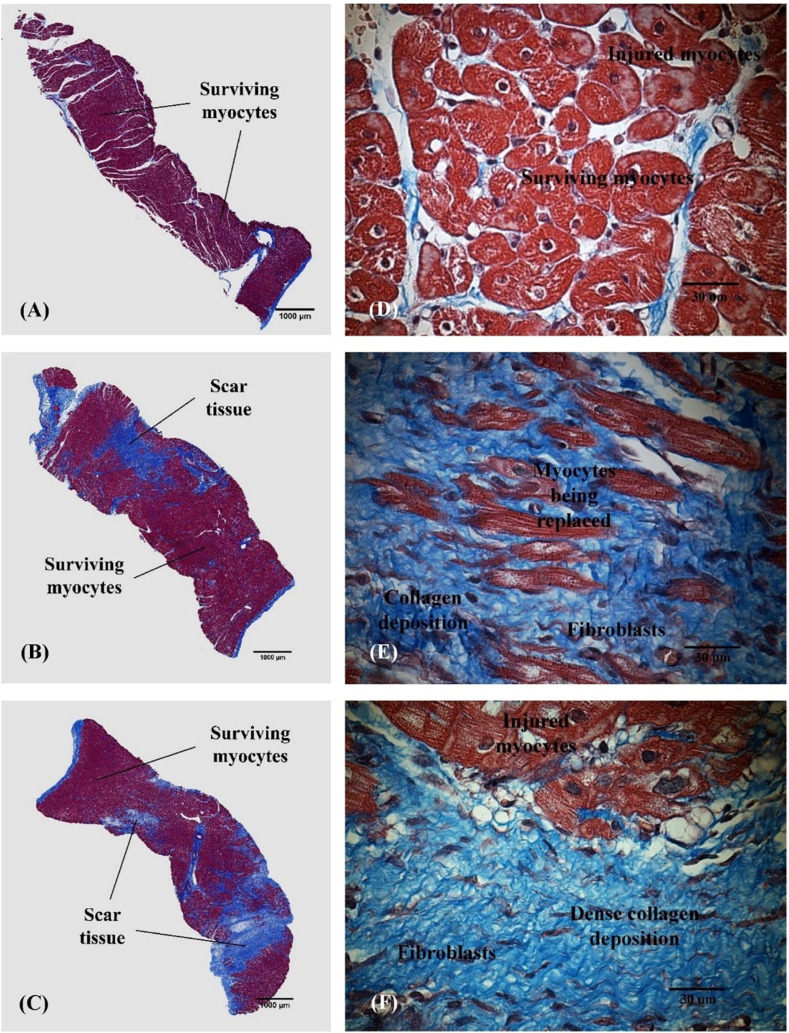
Representative histological images obtained from the area at risk in the anterior wall of the left ventricle in the SARP-b **(A,D)**, SARP-nb **(B,E)**, and control **(C,F)** groups. Masson’s trichrome *2x* and *60x*. Red = Viable myocardium, Blue = Fibrotic tissue.

Figure 5 shows histological images taken from the area at risk in representative animals from the SARP-b (Figures 5A, D), SARP-nb (Figures 5B, E), and control (Figures 5C, F) groups. The blue area is the myocardial infarction stained with Masson’s Trichrome. The fibrotic area in the SARP-b group (9.4% ± 1.4%) was significantly lower than in the SARP-nb group (34.2% ± 5.3%, *p* < 0.05) and the control group (44.6% ± 1.7%, *p* < 0.01). There was no statistically significant difference in fibrotic area between the SARP-nb and the control groups.

Our model simulations indicate that SARP significantly enhances the clearance or “washout” — of metabolites from ischemic regions, channeling the tracers into the cardiac chambers via Thebesian veins (Supplementary Figures S2–S5). This washout mechanism helps to remove potentially toxic substances that can accumulate during ischemia, thereby mitigating cellular damage. Moreover, the model simulations show that oxygenated blood from the femoral artery can be redirected back to the capillaries within the ischemic region (Supplementary Figures S1, S2).

## Discussion

The data from the present study in a STEMI swine model demonstrates that selective autoretroperfusion (SARP) preserves cardiac function and reduces myocardial infarct size significantly, possibly through a washout effect. The LVEF in the SARP-b group was >50% during retroperfusion and at the end of the study, compared to the control group with LVEF ∼40%. The area of infarction in the SARP-b group was ∼10% compared to ∼35% in the control group. Furthermore, and perhaps the most interesting finding of the study, was the progressive reduction in the concentration of lactate and other analytes during 10 min of retroperfusion, which points out to a washout effect of SARP.

Novel modalities of treatment to limit ischemia reperfusion injury (RI) in patients with acute myocardial infarction are urgently needed to combat morbidity and mortality following STEMI (Fröhlich et al., 2013; Bulluck et al., 2016; Li et al., 2019). In the present study, we validated the efficacy of short-term selective autoretroperfusion (SARP), which was implemented using our novel SARP catheter via the great cardiac vein in a setting of impending myocardial infarction, as an intervention against RI *prior* to complete restoration of blood flow through the arterial system. This preconditioning technique, which uses the animal’s own pulse pressure without the need of synchronized pumps, resulted in significant reduction of myocardial infarct size possibly through a washout effect of metabolic byproducts like H+ and lactate accumulating in the ischemic tissue during the occlusion.

Ischemic preconditioning is a protective technique that provides the myocardium with short periods of nonlethal ischemia and reperfusion before the ischemic insult (Murry et al., 1986). This protective mechanism has been demonstrated in several animal species and also in humans and may be part of some naturally occurring ischemic syndromes (Schott et al., 1990). It also enhances tolerance to lethal cell injury, protects against other end points of ischemia-reperfusion injury, and decreases apoptosis, a mechanism that can contribute to cell death after myocardial ischemia and reperfusion (Iliodromitis et al., 2007). Although we applied traditional preconditioning to all three groups of animals, which may have conferred certain beneficial effects, the area of infarction in the control group was significantly larger than in the SARP-b and SARP-nb groups. More importantly, unlike SARP, traditional preconditioning cannot be applied to STEMI patients once the arterial occlusion has occurred.

Coronary venous retroperfusion has been used in the past (Pratt, 1898; Beck and Stanton, 1948) to treat the ischemic myocardium. Due to the significant harm, however, caused by persistent application of arterial pressure to the venous circulatory bed (Hammond et al., 1967), this method of therapy was largely abandoned (Syeda et al., 2004). SARP, our proposed method of retroperfusion, is differentiated by the use of pressure-controlled delivery of arterial blood to the ischemic myocardial region just prior to PCI and for a brief period of time. This study demonstrated the benefits of a catheter-based, pressure-regulated (∼60 mmHg) approach of SARP to supply arterial blood to the ischemic zone without the use of synchronized pumps. Furthermore, a short-term administration of SARP in a swine model of anterior LV myocardial infarction, was sufficient to significantly reduce infarct size. Although the area of infarction with 10 min of retroperfusion was larger than the area of infarction with 30 min of retroperfusion [data from our previous study (4.7% ± 1.6%)]^26^, the values were not significantly different (*p* > 0.05).

Retroperfusion pressure (∼60 mmHg) as well as retroperfusion flow are two paramount factors that impact the reduction of myocardial edema and hemorrhage, and oxygen delivery to the ischemic territory, respectively. The efficacy of SARP relies in the ability to control the increase in pressure in the perfused venous territory preserving at the same time coronary artery inflow and cardiac venous drainage. SARP is possible because there exist many inter-venous connections (Kassab et al., 1985). In our study, pressure in the GCV during retroperfusion was 63.9 ± 4.6 mmHg in the SARP-b group. The LAD artery occlusion led to a significant decrease in arterial pressure, necessitating the use of norepinephrine for optimal retroperfusion pressure. In the SARP-nb group, the retroperfusion pressure was 39.2 ± 8.6 mmHg but since the balloon of the SARP catheter was deflated, the pressure was not transmitted towards the venous territory in the LV anterior wall. Additionally, controlled increased of pressure in the venous system increases collateral flow towards the ischemic region (Sato et al., 1996), due to the interconnections between the coronary sinus, the venous plexus and the Thebesian vessels (Kassab et al., 1985).

Diverse mechanisms of cardiac necrosis after ischemia-reperfusion injury that have been demonstrated include, reactive oxygen species, hemorrhage, and neutrophil accumulation (Kalogeris et al., 2012), microvascular spasm, edema, calcium overload, and mitochondrial dysfunction (Papageorgiou et al., 2018). The mechanisms behind the beneficial effects of retroperfusion, however, are not totally understood. Besides redistribution of flow as a mechanism of action for retroperfusion (De Maria et al., 2016), an increased washout of potentially hazardous byproducts from the microcirculation in the ischemic myocardium, seems to play a key role against reperfusion injury. Although the washout mechanism has been proposed previously (Chang et al., 1987; Mohl et al., 2018), to our knowledge, this is the first time that measurement of effluent blood samples corroborates the effect through SARP (Figure 3). The amount of several analytes measured during short-term retroperfusion when the LAD artery was still occluded, gradually returned to close-to-baseline levels, which prevented injury to the myocardium when reperfusion occurred. The ultimate beneficial effect of SARP was a substantial reduction in myocardial infarct size without myocardial injury from either reperfusion or SARP itself. Again, it is worth noting that effluent blood was not present in the control group, which supports the claim that blood from the femoral artery is redirected back to the capillaries and the occluded arteries within the ischemic territory during retroperfusion.

During acute myocardial infarction, dysfunction of myocardial cell membrane occurs, which leads to edema and leakage of cellular contents into the interstitial layer, with accumulation of toxic byproducts in the area affected by the lack of blood supply. Furthermore, the washout rate significantly decreases in the ischemic region (Chang et al., 1987). Ischemia leads to decrease in cell pH, excess hydrogen ions, and ATP depletion, causing calcium overload (Kalogeris et al., 2012). When blood supply is reestablished during reperfusion, the delivery of oxygen triggers a cascade of events that exacerbates myocardial injury. The antegrade delivery of oxygenated blood during reperfusion is higher in non-jeopardized areas of the myocardium (Chang et al., 1987) which may explain why, even with prompt reperfusion, salvage of the ischemic region is not always successful. On the other hand, when retroperfusion of oxygenated blood is established via the coronary veins, the delivery of oxygenated blood is more selective towards the ischemic myocardium (Chang et al., 1987), and at lower pressure than antegrade perfusion, more gently enhancing the washout of toxic metabolites in these regions. Furthermore, it has been hypothesized that the increase in pressure in the venous vessels triggers embryonic molecular pathways that may contribute to the salvage of the ischemic myocardium (Mohl et al., 2008).

The computer simulations offer some insights into the mechanisms of action of SARP in reducing infarct size. Specifically, retrograde perfusion not only facilitates the delivery of essential oxygen and nutrients but also ensures the removal of metabolic byproducts, thus creating a dual-effect mechanism of action of SARP in reducing infarct size. This interplay between the washout effect and the redistribution of oxygenated arterial blood is an important aspect of SARP. SARP significantly contributes to the reduction of infarct size as indicated by the experimental data, probably due to the combined effects of the removal of harmful metabolites and the supply of oxygen-rich blood. Simulation of the role of oxygen and nutrients in SARP remains for future studies.

Finally, deliverability is a common limitation for therapies targeting RI since they should be administered prior to reperfusion to truly precondition the coronary vasculature and myocardium. SARP provides an option to effectively deliver treatment since the device is relatively easy to navigate. The simplicity of the SARP catheter makes it attractive for utilization alone or in combination with other modalities of treatment like hypothermia, cell, or drug delivery.

### Limitations

There are several limitations associated with this study. First, the mathematical model treats the pressure in the GCV during retroperfusion as an input derived from experimental observations for both SARP-b and SARP-nb cases. This approach circumvents a broader challenge of 0D lumped parameter models in capturing wave reflection phenomena. Second, the model does not encompass oxygen transport mechanisms. This omission curtails the model’s applicability in predicting myocardial metabolism and functionality, especially in ischemia and/or retroperfusion. Third, the arterial-capillary-venous network model is constructed using historical experimental data from swine.

## Conclusion

PCI has significantly improved the outcome in acute myocardial infarction patients, but many still experience extensive myocardial damage due to reperfusion injury, leading to ventricular dysfunction and heart failure. Our study demonstrated that following coronary occlusion, a short (10 min) pretreatment with SARP *prior* to full restoration of antegrade flow greatly reduced myocardial infarct size in swine. The implications of this study suggest that myocardial injury due to reperfusion can be mitigated potentially by a washout mechanism by changing the manner of flow restoration and delaying the opening of the occluded artery for a brief period of time while SARP is implemented and the patient is prepared for PCI.

## Data Availability

The original contributions presented in the study are included in the article/Supplementary Material, further inquiries can be directed to the corresponding author.
