# Supplementary material for: Preconditioning with selective autoretroperfusion: In vivo and in silico evidence of washout hypothesis
Source: Front Bioeng Biotechnol. 2024 May 10;12:1386713. doi: 10.3389/fbioe.2024.1386713 (PMC11117169; doi:10.3389/fbioe.2024.1386713)
Supplement: Supplementary file 1 [file DataSheet1.docx]

**SUPPLEMENTARY MATERIALS**

**Methods**

***Electrocardiogram***

Electrocardiographic leads were placed on the animals’ limbs to monitor the electrical activity of the heart and the heart rate. The data were analyzed using the ECG analysis software in LabChart Pro (ADInstruments, Colorado Springs, CO), with preset settings for swine. Tables S1 and S2 show the heart rate and ST-segment changes, respectively, in each group at different time points.

***Echocardiography***

Transesophageal echocardiograms were obtained at baseline (5 minutes before premedication with Amiodarone), during LAD occlusion (5 minutes before initiation of retroperfusion), during retroperfusion (5 minutes before initiation of reperfusion), and at 4 weeks. An EPIQ 7C ultrasound system (Philips, Andover, MA) with an X8-2t transducer was used. Four-chamber 2D and 3D echocardiographic images were acquired with the animals placed in the supine position. Left ventricular ejection fraction (LVEF), end-diastolic diameter (LVEDD), end-systolic diameter (LVESD), end-diastolic wall thickness (LVEDWT), and end-systolic wall thickness (LVESWT) were calculated offline using QLAB 10.8 (Philips Healthcare, Andover, MA). LVEF was calculated from 4-chamber longitudinal views, using the Simpson’s method. The parasternal long axis views were used to determine LV diameter. The wall thickness was the average value of six measurements taken on short axis views at the anterior, anteroseptal, anterolateral, posterior, posteroseptal, and posterolateral LV wall. Additionally, we calculated LV global and segmental longitudinal strain from long axis view images, using QLAB 10.8 aCMQ (automated cardiac motion quantification).

***Blood Sampling***

Retroperfusion effluent blood samples were collected to measure several analytes like pH, glucose, lactate, potassium, sodium, and calcium. The retroperfusion effluent samples (1 mL) were obtained every 2 minutes via the lumen of the PTCA balloon catheter occluding the LAD artery in the SARP-b and SARP-nb groups. In the control group, blood through the PTCA catheter was impossible to withdraw. Central venous blood was also obtained at different time points (5 minutes before premedication with Amiodarone, 5 minutes before the end of ischemia, immediately after reperfusion, and at 4 weeks) to determine cardiac troponin (cTnI) levels. Samples were analyzed using i-STAT test cartridges and an i-STAT Alinity analyzer (Abbott Laboratories, Abbott Park, IL).

***Heart Preparation***

After 4 weeks, the heart was arrested in diastole with potassium chloride injected through the jugular vein while the animal was well anesthetized. The heart was excised and transported to the lab in 0.9% sodium chloride. Transmural biopsies were taken from the area at risk in the LV for histological analysis. The LAD, left circumflex, and right coronary arteries were cannulated with Tygon tubing and perfused with saline to flush out the blood. The LAD artery was ligated with silk suture below the second diagonal branch, at the same site where the PTCA balloon catheter was inflated during the SARP procedure. The coronaries were then perfused with 1% Evans blue, 15 mL, to demarcate the non-infarcted myocardium (blue). The LV was then cut into 7-8 slices (~10 mm thick) from apex to base. The slices were further stained with 1% 2,3,5-triphenyltetrazolium chloride (TTC) at 37°C, fixed in 10% buffered formalin, and scanned. In viable myocardium, the tissue appears dark red (area at risk) because TTC is converted by dehydrogenases to a red formazan pigment. In necrotic myocardium, on the other hand, this does not occur due to the loss of dehydrogenases which render the tissue white. The size of the infarcted myocardium was measured manually, using ImageJ software (National Institutes of Health), and expressed as a percentage relative to the area at risk. Similarly, the area at risk was measured and expressed as a percentage relative to the LV area. For the calculations, the mean values of both sides of each slice were used.

**Mathematical Model**

***Continuous Release of Indicators***

Figure S2 shows the distribution of indicators following their continuous release at two locations within the ischemic region: an artery of order 7 and a vein of order -7 (referenced as ‘art7’ for the artery and ‘ven-7’ for the vein in Figure S1), illustrating the dispersal pattern resulting from the release of 1,000 indicators per instance under two different conditions (control vs. SARP). When released in the ‘art7’, the concentration of the indicators as a percentage is ~77.8% in the Thebesian veins with SARP, indicating a SARP-enhanced washout effect that indicators (metabolites) move away from the release point into the chambers through Thebesian veins. In the control group, all indicators are stuck in vessels that are closely connected to the release point (‘art7’) with no indicators present in the capillaries or Thebesian veins, suggesting nearly no washout in the control condition.

Upon release at the vein of order -7 (ven-7), the indicators (representing red blood cells) demonstrate the ability to traverse from the point of release back to the capillaries within the ischemic region under both SARP and control conditions. With SARP, the indicators’ concentration within the Thebesian veins approximates 38.8%, whereas in the control condition, it is approximately 2.5% (*p*<0.001). In the capillaries, the indicators’ concentration reaches about 35.4% when SARP is applied, compared to 24.1% in the absence of SARP in the control group (*p*<0.01). It is particularly noteworthy that under the control condition, the blood that travels into the capillaries is deoxygenated. Conversely, with SARP, the blood entering the capillaries is oxygenated.

Figure S3 shows the distribution of indicators (metabolites) following their continuous release at arterioles of order 1 (referenced as ‘art1’ in Figure S1) under these two different conditions (control vs. SARP). Importantly, the indicators’ concentration within the Thebesian veins reaches about 80.0% when SARP is applied, compared to only 4.2% in the absence of SARP in the control group (*p*<0.001).

***One-Time Release of Indicators***

Figures S4 and S5 show the distribution of indicators after 10 minutes following a one-time release of 1,000 indicators at different locations. Upon release at the artery of order 7 (art7) and with SARP, approximately 77.8% indicators (metabolites) move away through the Thebesian veins, 11.2% and 10% stay in the artery of order 1 and capillaries, respectively. In the control group, 20% stay in the artery of order 7 and 80% move into the artery of order 9 (Figure S4). Upon release at the artery of order 1 (art1, Figure S5), approximately 80.0% indicators (metabolites) move away through the Thebesian veins with SARP while only 4.2% in the control case (*p*<0.001).

**Table S1. Heart rate at different time points within each group.**

| **Heart rate (bpm)** | **SARP-b** | **SARP-nb** | **Control** |
| --- | --- | --- | --- |
| Baseline | 87 ± 3 | 74 ± 6 | 80 ± 5 |
| Preconditioning | 81 ± 2 | 85 ± 7 | 78 ± 4 |
| Ischemia | 93 ± 5 | 79 ± 5 | 80 ± 5 |
| Retroperfusion | 105 ± 5^b,c^ | 99 ± 6^a^ | 85 ± 5 |
| Immediate reperfusion | 101 ± 3^b,d^ | 91 ± 6 | 85 ± 4 |
| Four weeks reperfusion | 84 ± 3 | 76 ± 5 | 77 ± 4 |

With respect to baseline in their corresponding group, the *p*-values were, ^a^*p*<0.05, ^b^*p*<0.01. With respect to Control, the *p*-values were, ^c^*p*<0.05, ^d^*p*<0.01.

**Table S2. ST-segment height at different time points within each group.**

| **ST-segment elevation (mV)** | **SARP-b** | **SARP-nb** | **Control** |
| --- | --- | --- | --- |
| Baseline | 0.0135 ± 0.0031 | 0.0109 ± 0.0053 | 0.0188 ± 0.0042 |
| Preconditioning | 0.0272 ± 0.0080 | 0.0156 ± 0.0041 | 0.0217 ± 0.0062 |
| Ischemia | 0.1064 ± 0.0107^c^ | 0.0939 ± 0.0061^c^ | 0.1049 ± 0.0304^a^ |
| Retroperfusion | 0.0675 ± 0.0054^c^ | 0.1180 ± 0.0088^c,e^ | 0.1725 ± 0.0346^b,d^ |
| Immediate reperfusion | 0.0514 ± 0.0068^c^ | 0.1054 ± 0.0155^b,d^ | 0.1157 ± 0.0204^b,d^ |
| Four weeks reperfusion | 0.0103 ± 0.0026 | 0.0150 ± 0.0028 | 0.0152 ± 0.0058 |

With respect to baseline in their corresponding group, the *p*-values were, ^a^*p*<0.05, ^b^*p*<0.01, ^c^*p*<0.001. With respect to SARP-b, the *p*-values were, ^d^*p*<0.05, ^e^*p*<0.01.

**Table S3.** Diameter, length, and branching ratio of coronary vessels. $D_{\exp}$, $L_{\exp}$, and $B_{\exp}$ are measured in pigs; $D_{\mathrm{num}}$, $L_{\mathrm{num}}$, and $B_{\mathrm{num}}$ are used in the present numerical network. Thebesian veins are representatives (diameter $=$10 µm, length $=$100 µm) aiming to account for the ratio of Thebesian outflow to coronary sinus outflow which is about 5-15% (6% in the present model). ac = post-arteriole capillary, cp = capillary, cc = capillary cross-connection, cv = pre-venule capillary.

| Order | $D_{\exp}$ (µm) | $D_{\mathrm{num}}$ (µm) | $L_{\exp}$ (µm) | $L_{\mathrm{num}}$ (µm) | $B_{\exp}$ | $B_{\mathrm{num}}$ |
| --- | --- | --- | --- | --- | --- | --- |
| 11 | 3276 | 3276 ± 0 | 52900 | 52900 ± 0 | 1 | 1 |
| 10 | 1695 ± 481 | 1695 ± 0 | 23900 ± 20100 | 23900 ± 0 | 8 | 8 |
| 9 | 778 ± 154 | 778 ± 0 | 11800 ± 10400 | 11800 ± 0 | 6 ± 0.25 | 6 |
| 8 | 460 ± 42.7 | 460 ± 0 | 5580 ± 3470 | 5580 ± 0 | 3.52 ± 0.21 | 3.52 |
| 7 | 290 ± 30.1 | 290 ± 0 | 3680 ± 2040 | 3680 ± 0 | 2.99 ± 0.23 | 2.99 |
| 6 | 138 ± 24.7 | 138 ± 0 | 1420 ± 1040 | 1377 ± 172 | 4.08 ± 0.40 | 4.08 |
| 5 | 65.2 ± 11.6 | 65.2 ± 5.54 | 508 ± 351 | 508 ± 409 | 4.23 ± 0.71 | 4.23 |
| 4 | 30.1 ± 5.4 | 30.1 ± 4.47 | 276 ± 163 | 276 ± 161 | 2.99 ± 0.95 | 2.99 |
| 3 | 17.1 ± 1.7 | 17.1 ± 1.83 | 149 ± 94 | 149 ± 78.5 | 2.44 ± 1.10 | 2.44 |
| 2 | 12.1 ± 1.1 | 12.1 ± 1.08 | 136 ± 88 | 136 ± 87.1 | 3.17 ± 1.67 | 3.17 |
| 1 | 9 ± 0.73 | 9 ± 0.70 | 115 ± 66 | 115 ± 71 | 2.63 ± 1.59 | 2.63 |
| ac | 6.2 ± 1.1 | 6.2 ± 0.61 | 52.0 ± 32.3 | 51.5 ± 18.7 | - | - |
| cp | 5.7 ± 1.2 | 5.7 ± 0.70 | 54.5 ± 43.0 | 55.6 ± 25.0 | - | - |
| cc | 5.5 ± 1.4 | 5.5 ± 0.68 | 21.1 ± 15.5 | 21.4 ± 9.1 | - | - |
| cv | 7.0 ± 1.2 | 6.9 ± 0.70 | 45.0 ± 30.5 | 45.3 ± 17.4 | - | - |
| -1 | 10.6 ± 1.6 | 10.6 ± 0.82 | 79 ± 54 | 79 ± 48.77 | 3.07 ± 1.39 | 3.07 |
| -2 | 16.5 ± 2.7 | 16.5 ± 1.47 | 92 ± 65 | 92 ± 58.92 | 3.22 ± 1.22 | 3.22 |
| -3 | 29.6 ± 3.2 | 29.6 ± 3.17 | 117 ± 71 | 117 ± 61.61 | 2.65 ± 0.83 | 2.65 |
| -4 | 57.5 ± 11.8 | 57.5 ± 8.53 | 350 ± 277 | 350 ± 204.14 | 3.89 ± 1.05 | 3.89 |
| -5 | 117 ± 18.7 | 117 ± 9.94 | 698 ± 649 | 698 ± 562.02 | 3.25 ± 0.82 | 3.25 |
| -6 | 205 ± 25.6 | 205 ± 0 | 1260 ± 1130 | 1260 ± 0 | 2.89 ± 0.70 | 2.89 |
| -7 | 317 ± 32.7 | 317 ± 0 | 2080 ± 1870 | 2080 ± 0 | 3.19 ± 0.70 | 3.19 |
| -8 | 488 ± 46.5 | 488 ± 0 | 3620 ± 3190 | 3620 ± 0 | 3.11 ± 0.62 | 3.11 |
| -9 | 773 ± 62.4 | 773 ± 0 | 6070 ± 5110 | 6070 ± 0 | 3 ± 0.52 | 3 |
| -10 | 1165 ± 88.2 | 1165 ± 0 | 10200 ± 8320 | 10200 ± 0 | 3.63 ± 0.5 | 3.63 |
| -11 | 1804 ± 464 | 1804 ± 0 | 25800 ± 24200 | 25800 ± 0 | 16 ± 2 | 16 |
| -12 | 5919 | 5919 ± 0 | 71900 | 71900 ± 0 | 1 | 1 |

**Table S4.** Parameters describing passive properties of vessels. Parameters across all vessel orders of the entire network are obtained by smooth spline fitting of the data listed below.

| Vessel order | $A_{p}$ ($\mu$m) | $B_{p}$ ($\mu$m) | $\varphi_{p}$ (mmHg) | $C_{p}$ (mmHg) |
| --- | --- | --- | --- | --- |
| 0 | 3.20 | 2.43 | 0 | 19.28 |
| 1 | 4.51 | 3.59 | 0 | 17.24 |
| 5 | 35.02 | 10.9 | 0.61 | 20.11 |
| 6 | 51.38 | 16.41 | 1.88 | 14.23 |
| 6 | 85.53 | 32.17 | 1.64 | 21.24 |
| 7 | 133.52 | 51.6 | 0.96 | 23.54 |
| 10 | 829.6 | 401.52 | 30.39 | 3.35 |

**Table S5.** Parameters used in the peripheral circulation.

| Parameter | Units |  |
| --- | --- | --- |
| $R_{\mathrm{ao}}$ | mmHg ms/mL | 2.25 |
| $R_{\mathrm{ven}}$ | mmHg ms/mL | 6.75 |
| $R_{\mathrm{mv}}$ | mmHg ms/mL | 5.0 |
| $R_{\mathrm{fm}}$ | mmHg ms/mL | 94.5 |
| $R_{per\_p}$ | mmHg ms/mL | 135 |
| $R_{per\_d}$ | mmHg ms/mL | 693 |
| $C_{\mathrm{art}}$ | mL/mmHg | 1.067 |
| $C_{\mathrm{ven}}$ | mL/mmHg | 106.67 |
| $C_{\mathrm{fm}}$ | mL/mmHg | 0.32 |
| $C_{\mathrm{per}}$ | mL/mmHg | 2.35 |
| $V_{\mathrm{LA}}$ | mL | 31 |
| $V_{\mathrm{LV}}$ | mL | 118 |
| $V_{\mathrm{art}}$ | mL | 1880 |
| $V_{\mathrm{fm}}$ | mL | 33 |
| $V_{\mathrm{per}}$ | mL | 168 |
| $V_{\mathrm{ven}}$ | mL | 3140 |
| $V_{LA0}$ | mL | 10 |
| $V_{LV0}$ | mL | 10 |
| $V_{art0}$ | mL | 1180 |
| $V_{fm0}$ | mL | 10 |
| $V_{per0}$ | mL | 10 |
| $V_{ven0}$ | mL | 1980 |

**Table S6**. Parameters used in the time-varying elastance models. LA, left atrium; LV, left ventricle.

| Parameter | Units |  |
| --- | --- | --- |
| $T_{max,LA}$ | ms | 150 |
| $A_{\mathrm{LA}}$ | mmHg | 58.67 |
| $B_{\mathrm{LA}}$ | mL^-1^ | 0.05 |
| $\tau_{\mathrm{LA}}$ | ms | 25 |
| $E_{es,LA}$ | mmHg/mL | 80 |
| $T_{max,LV}$ | ms | 190.4 |
| $A_{\mathrm{LV}}$ | mmHg | 140 |
| $B_{\mathrm{LV}}$ | mL^-1^ | 0.024 |
| $\tau_{\mathrm{LV}}$ | ms | 25 |
| $E_{es,LV}$ | mmHg/mL | 400 |

**
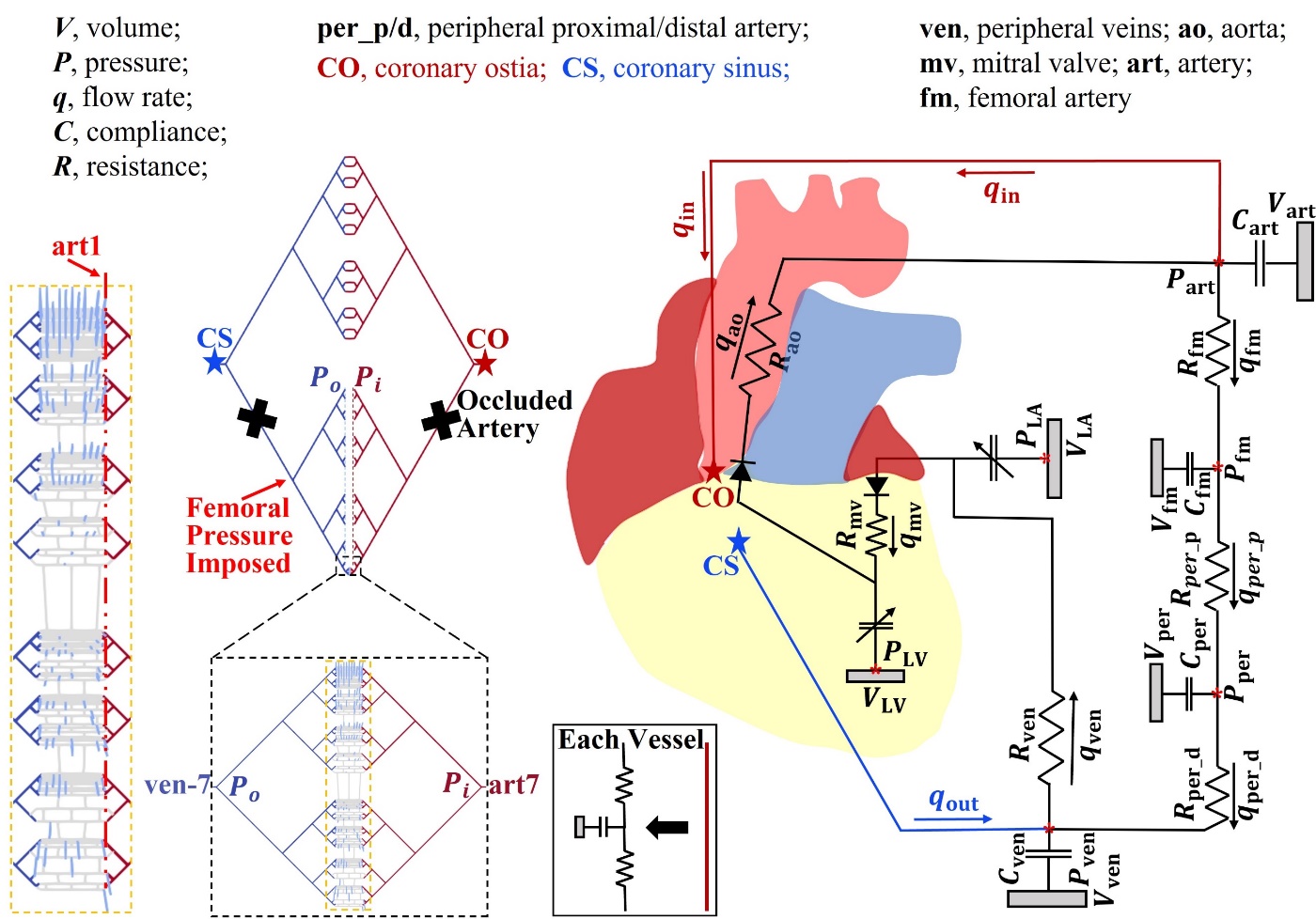
**

**Figure S1.** Schematic of the closed-loop computational modeling framework. Inset with black dashed border shows the coronary microvascular network containing coronary arteries, capillaries, and veins. Inset with solid border shows the representation of each vessel by a three-element Windkessel model consisting of two resistances and a capacitance. The black cross indicates the occluded coronary artery and the distal great cardiac vein.

**
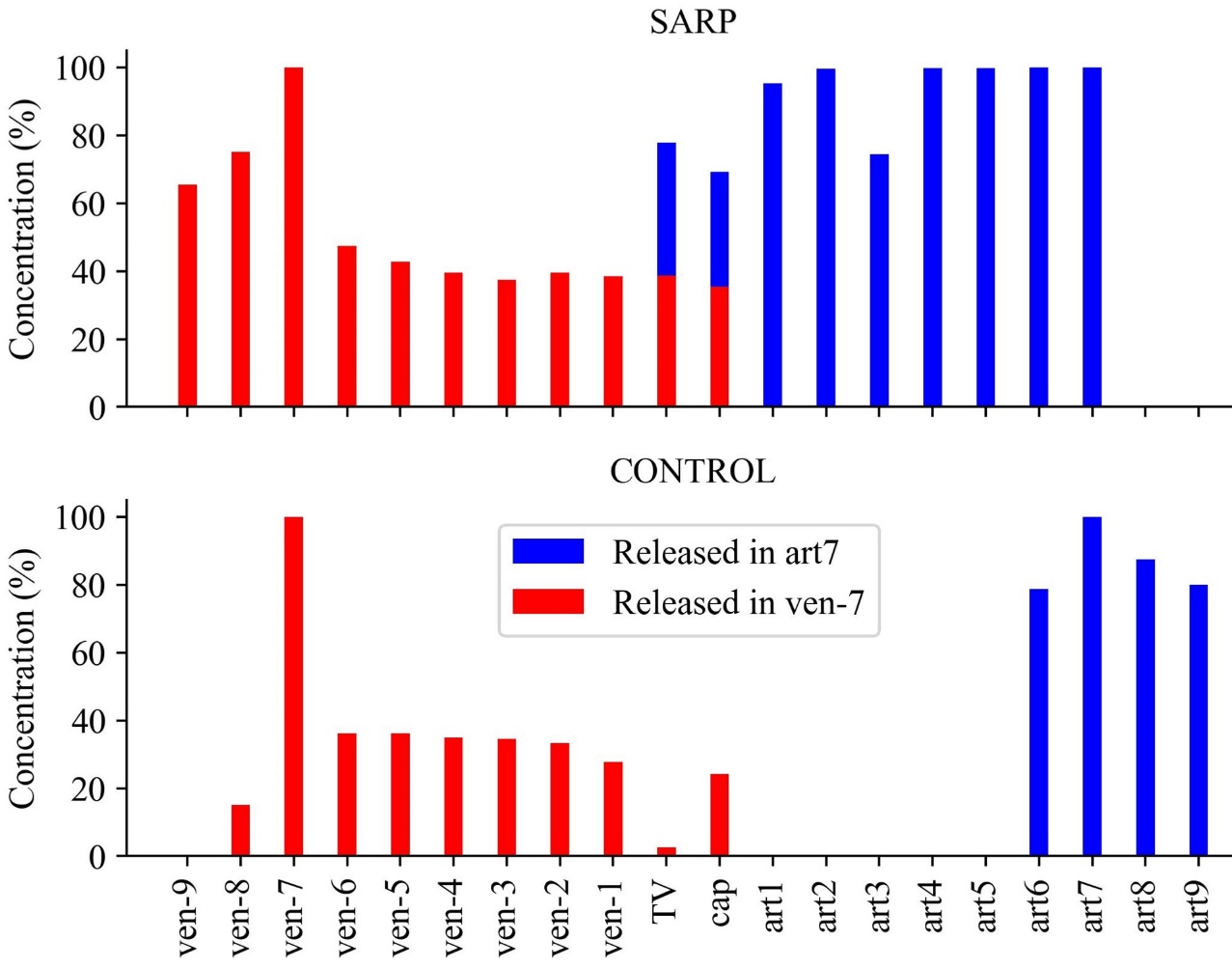
**

**Figure S2.** Distribution of indicators during a continuous (permanent) release at the artery of order 7 and the vein of order -7 (referenced as ‘art7’ for the artery and ‘ven-7’ for the vein in Figure S1). This figure illustrates the ongoing dispersal pattern resulting from the release of 1,000 indicators per instance. Art9~1, artery of order 9~1; ven-1~-9, vein of order -1~-9; cap, capillary; TV, Thebesian veins.


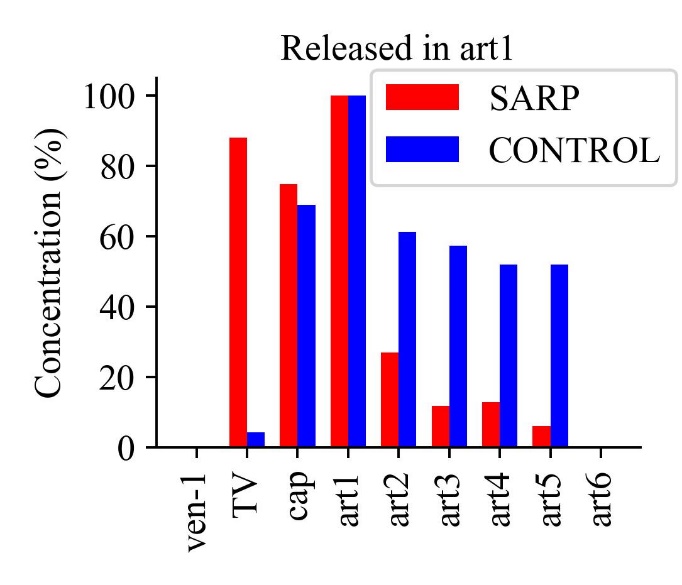


**Figure S3.** Distribution of indicators during a continuous release (1,000 indicators per instance) at the artery of order 1 (referenced as ‘art1’ in Figure S1). This figure illustrates the ongoing dispersal pattern resulting from the release of 1,000 indicators per instance. Art6~1, artery of order 6~1; ven-1, vein of order -1; cap, capillary; TV, Thebesian veins.


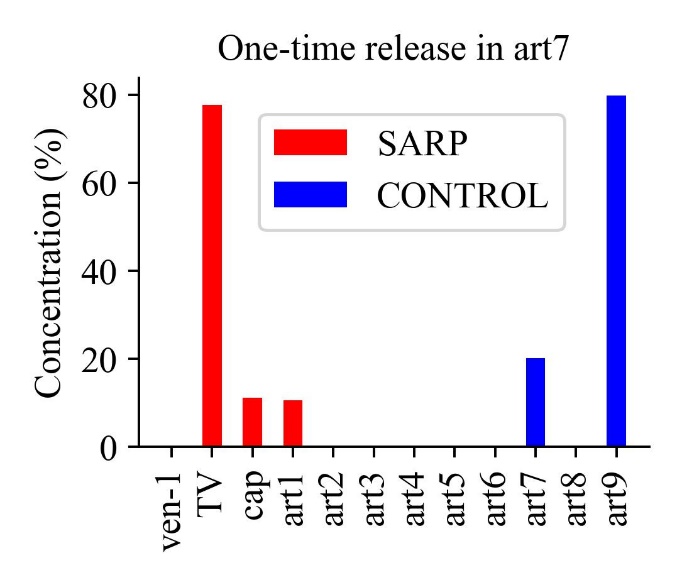


**Figure S4.** Distribution of indicators after 10 minutes following a single (one-time) release of 1,000 indicators at the artery of order 7 (referenced as ‘art7’ in Figure S1). Art9~1, artery of order 9~1; ven-1, vein of order -1; cap, capillary; TV, Thebesian veins.


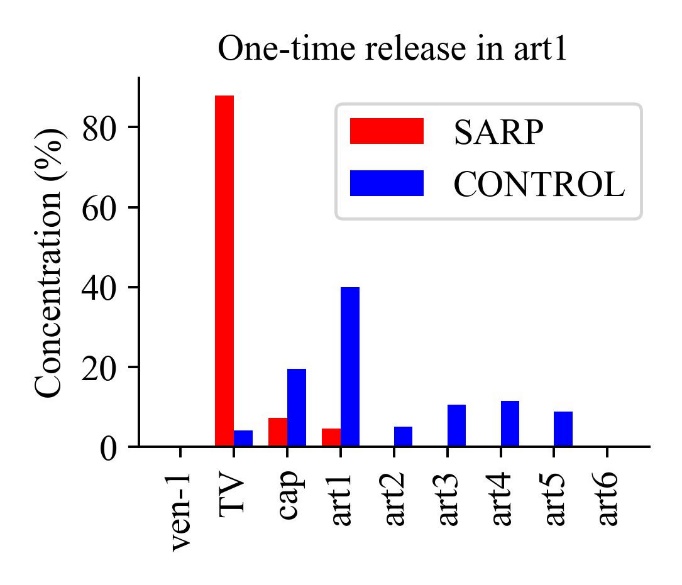


**Figure S5.** Distribution of indicators after 10 minutes following a single (one-time) release of 1,000 indicators at the artery of order 1 (referenced as ‘art1’ in Figure S1). Art6~1, artery of order 6~1; ven-1, vein of order -1; cap, capillary; TV, Thebesian veins.
